# Dr. Stewart’s Account of the Epidemic Influenza Prevailing at Paris; Known There under the Name of “La Grippe”

**Published:** 1842-06-04

**Authors:** F. Campbell Stewart

**Affiliations:** Paris


### MEDICAL EXAMINER.

NEW SERIES.

No. 23.] PHILADELPHIA, JUNE 4, 1842. [Vol. I.

*An account of the Epidemic Influenza prevailing at Paris; known there under the name of "la Grippe."* By F. Campbell Stewart, M. D.

Since the year 1830, Paris has been subject to the recurrence of an epidemic catarrh which has made its appearance at four several and distinct epochs, and the nature of which has varied with atmospheric changes, preserving, however, in all cases, a decided predilection for the mucous membranes, which are always the principal seat of the malady.

The term *Grippe* (from the French verb *agripper*,—to seize suddenly and violently, to clutch,) was applied to the disease about the close of the last century, previously to which time it had been known in France as le *tac*, le *horion*, la *dando*, &c. Its present French synonymes are, la *follette*, la *baraquette*, la *petite-poste*, le *petit-courrier*, &c. &c.

It would be inferred from the term by which it is most commonly designated, (Grippe,) that the invasion of the disease is very sudden. This is not generally the case, however, and though its early effects are prostrating and distressing in the extreme, it frequently requires three days for the disease to become fully developed.

The present epidemic, although more general, is by no means of so serious a nature as was that of 1837, the termination of which was frequently fatal, whereas, at present, it is productive of only a temporary, though often most serious indisposition. Its exciting cause is undoubtedly atmospheric vicissitudes, which have been frequent during the last month or six weeks, both as regards sudden changes of temperature, and transitions from dry to wet, with the prevalence, for the most part, of a cold, damp, westerly and south-westerly wind.

The approach of the disease is announced by a general debility and lassitude, with pain in the back and extremities, fulness of the head, loss of appetite, yawning, restlessness, anxiety, nervousness, and most of the prodromes of intermittent fever. On the second or third day there are fever and cephalalgia, with an aggravation of the other symptoms. The throat then becomes sore, the tonsils are swollen, and there is much irritation about the larynx and pharynx, extending in some instances to the trachea and bronchiæ, and occasioning a constant and irritating cough. The local inflammation is trifling, and its existence only known by a slight redness of the mucous membrane of the mouth and throat, and the little swelling and pain that usually accompany the disease. Generally speaking, the patient is not obliged to keep to his bed, but sometimes the distress is so great as to preclude the possibility of making the slightest exertion, or of retaining any other than the recumbent posture.

The duration of the disease, when left to itself, is usually from five to eight days, when it declines, and generally terminates in a free coryza.

The treatment may be for the most part expectant, and little is done by the French physicians more than to prescribe one or two warm baths, and have them followed by demulcent tisanes.

In such cases, amongst our countrymen, as have come under my own care, I have generally succeeded in abridging the duration of the disease, and relieving the most distressing symptoms by simple means. Commencing with a Dover's powder, administered at the earliest period, I direct a small dose of the blue mass to be taken for two consecutive nights, and followed in the mornings after by a Seidlitz powder, or some simple saline laxation. Where there is much oppression, or the subject is of a full, plethoric habit, the loss of eight or twelve ounces of blood alone will frequently prove sufficient to arrest the progress of the complaint. As a general rule, however, venesection is contra-indicated.

Occasionally the irritation extends to the mucous membrane of the intestines, and instead of costiveness, which generally exists, we have a slight diarrhœa. This is easily checked, however, by combining a little opium with the blue mass. In these cases I give a pill of the following composition every three hours :—

| | |
|---|---|
| ℞ Mass. hydrarg., | gr. xxiv. |
| Pulv. opii, | vi. |
| " scillæ, | xiii. |

To be made into twelve pills.

From the foregoing account of the Influenza, or Grippe, of Paris, every practitioner will at once perceive that it differs but little from the severer forms of the spring and autumn catarrhs of our northern and eastern states. No treatment is absolutely required, and when recourse is had to medicines such only should be selected as are of the mildest and most simple kind.

This disease has now become so common, and of such frequent recurrence in Paris, that it should be considered as rather endemic to the place than as a casual epidemic. It rarely, however, becomes so general as it has been during the present season, when scarcely an individual has escaped its influence. No profession, age, nor sex has been spared, and whole families are ill with it at the same time.

As it is not a very serious malady, few of the poorer classes are willing to subject themselves to a hospital régime, and hence they rarely resort to these public institutions when they have nothing else to complain of. The hospitals, however, present hundreds of cases of it amongst the surgical and medical patients, who were inmates previously to the invasion of the epidemic, and who have not escaped its influence. So little do the medical men attached to the hospitals think of it, however, that they rarely suspend a course of treatment or postpone an operation on its account.

The term *Grippe*, as applied to the disease which at present afflicts the population of Paris, like many others admitted into the medical vocabulary, conveys no intimation whatever of the nature of the disease which it is intended to designate; it was appropriate to the epidemic of 1837, when persons were suddenly seized with the disease, frequently whilst walking, or riding, and reduced to such an instant state of prostration as to require the most powerful stimulant treatment; but the present catarrh differs widely from that of the period just named, and there is no reason for the application of so unsuitable a term, other than that it is of common and universal acceptation.

*Paris, April,* 1842.

